# High-resolution metabolic profiling of the effects of early-life stress after peripheral nerve injury

**DOI:** 10.1016/j.jpain.2026.106270

**Published:** 2026-03-31

**Authors:** Kayleigh A. Rodriguez, Mary Grace Bishop, Laura R. Osborn, Dakota Redling, James D. Sikes, Craig Porter, Laxmi Yeruva, Kimberly E. Stephens

**Affiliations:** a Arkansas Children’s Research Institute, 13 Children’s Way, Little Rock, AR 72202, United States; b Department of Neuroscience, College of Medicine, University of Arkansas for Medical Sciences, 4301 W. Markham St, Slot 847, Little Rock, AR 72205, United States; c Department of Pediatrics, College of Medicine, University of Arkansas for Medical Sciences, 3 Children’s Way, Little Rock, AR 72202, United States; d Microbiome and Metabolism Research Unit, Arkansas Children’s Nutrition Center, US Department of Agriculture, Agricultural Research Service, 15 Childrens Way, Little Rock, AR 72202, United States

**Keywords:** Early-life stress, Metabolism, Nerve injury, Chronic pain, Somatosensory development

## Abstract

Current evidence supports an association between chronic pain and the promotion of metabolic disorders, which can be further exacerbated by environmental factors. Early-life stress is an environmental risk factor that is well known to influence pain susceptibility. However, the maladaptive effects of early life stress on metabolism in the setting of chronic pain is unknown. In this study, we used the well-established limited bedding model of early life stress and subjected male and female adult offspring and controls to metabolic and behavioral profiling before and after peripheral nerve injury. We provide continuous, two-week, high-resolution profiling of animal food and water intake, physical movement, and energy expenditure data to study the effects of nerve injury-induced pain on metabolic outcomes in adult rats following exposure to early life stress. Additionally, we differentiate between dark and light cycles, which is crucial for understanding circadian rhythms in rodents. All offspring are assessed for baseline and post-injury mechanical sensitivity. We find that early life stress increases mechanical hypersensitivity following peripheral nerve injury in female offspring and reduces energy expenditures after peripheral nerve injury in male offspring. Our data provide valuable insights into metabolic and behavioral responses following nerve injury and contribute to understanding chronic pain development in both male and female rats. However, future studies are needed to understand the impact of our findings toward the promotion of metabolic disorders observed clinically.

## Perspective

Mounting evidence supports associations between chronic pain and the promotion of metabolic disorders. We provide comprehensive metabolic assessments of the impact of ELS in the setting of nerve-injury induced pain to deepen our understanding of the mechanisms by which ELS influences pain sensitivity and metabolic outcomes after nerve injury.

## Introduction

Mounting evidence supports an association between chronic pain and the promotion of metabolic disorders including weight gain and loss of fat free mass.^1–3^ Indeed, altered levels of carbohydrate, lipid, protein and nucleic metabolites, which are involved in key cellular process and the trajectory of individual health, are well documented in individuals living with chronic pain.^1,3^ While the development of chronic pain and its association with metabolic conditions is complex and multifactorial, experimental pain studies in healthy adults demonstrate that pain alone is sufficient to induce maladaptive metabolic changes.^4^ Furthermore, nerve injury is a metabolic stressor that increases energy requirements and activates metabolic processes to facilitate pain and inflammation.^5,6^ However, it is unknown whether the long-term impact of pain-promoting metabolic changes are further exacerbated by the presence of environmental factors that influence both pain perception and physiological regulation.

During critical periods of neurodevelopment, an adverse environment can disrupt the maturation of somatosensory pathways, leading to long-term alterations in pain processing and regulation.^7–10^ Indeed, adverse environmental factors (e.g., socioeconomic status, household violence, early-life stress (ELS)) promote increased pain sensitivity and increased susceptibility to chronic pain conditions across diverse populations.^11,12^ ELS exerts multifaceted impacts on both metabolism and pain sensitivity, potentially creating a complex interplay to promote chronic pain. Stress alone disrupts critical metabolic processes and energy metabolism, including glucose metabolism, insulin sensitivity, and lipid metabolism.^13^ These dysregulated metabolic processes promote chronic low-grade inflammation which sensitize pain receptors to increase pain sensitivity.^14,15^ In addition, altered glucose metabolism and insulin signalling impair nerve function and promote peripheral neuropathies.^16^

ELS dysregulates the hypothalamic-pituitary-adrenal (HPA) axis which alters cortisol secretion and disrupts the body's stress response and impairs the ability to adapt to subsequent stressors.^17^ Elevated cortisol can suppress protein synthesis that is crucial for tissue repair and regeneration. Additionally, ELS can prime the immune system to react more strongly to injury, promoting increased inflammation at the site of damage and hindering healing.^18^ Disruptions of normal metabolic processes in response to insults such as nerve injury can impose challenges on recovery and exacerbate chronic pain. However, the literature lacks studies that examine the relationship between ELS, metabolic dysregulation, and chronic pain. In this study, we use high-resolution metabolic and behavioral profiling to study the effects of ELS on metabolic outcomes in adult male and female rats following sciatic nerve injury. Because sex-specific metabolic changes have been reported after nerve injury,^19^ we analyze and report findings separately for males and females. A comprehensive metabolic assessment of the impact of ELS in the setting of nerve-injury induced pain could lead to a deeper understanding of the mechanisms by which stress influences pain development and alters metabolic outcomes after nerve injury.

## Methods

### Animals

Timed-pregnant, nulliparous Sprague Dawley rats (Charles River Laboratories) were individually housed in polycarbonate cages with sawdust bedding upon arrival on gestational day 18. All animals were housed in temperature (22 ± 1°C) and humidity (50% ± 10%) controlled rooms with 12-hour light (0700–1900) and dark (1900 – 0700) cycle and were given access *ad libitum* to standard rodent chow (Labdiet Prolab RMH 1800) and water. All procedures involving animals were reviewed and approved by the Institutional Animal Care and Use Committee at the University of Arkansas for Medical Sciences and performed in accordance with the Guide for the Care and Use of Laboratory Animals published by the National Institutes of Health and ethical guidelines of the International Association for the Study of Pain. The public was not involved in the study’s conceptualization, design, or dissemination of results. All animal procedures and outcomes assessed were done in a blinded manner.

### Early-life stress paradigm

We implemented the Chronic Early life Stress (CES) model as detailed by Molet and colleagues.^20^ Upon arrival, dams were monitored twice daily for delivery. The day of birth was designated as postnatal day (PND)0. On PND2, pups were briefly separated from the dams for sexing and randomization. Dams were randomly assigned to an impoverished cage (CES group) or standard cage (STD group) (Fig. 1). Each dam assigned to the CES group was placed in a cage with a small amount of sawdust bedding which was separated from the animals by an aluminium mesh (McNichols Co, Tampa FL) placed 2.5 cm above the cage floor. One half of one paper towel was provided to each dam for nesting material. Each dam assigned to the STD group was placed in a cage with the standard amount of sawdust bedding and a whole paper towel for nesting material. To minimize genetic effects, all pups were sexed by anogenital distance and randomly allocated to a dam. Litters were culled to equal sizes (n=12) with a final sex ratio of approximately 1:1. Litters were left undisturbed from PND2 through PND9. On PND10, each litter was transferred to a clean, standard cage. To minimize disturbances to the litters, these animals were held in a dedicated room with all care provided by a single person from birth through weaning. Standard husbandry practices resumed after weaning on PND21.

### Establishment of pain model

Chronic constriction Injury (CCI) surgery to the sciatic nerve was performed as previously described on PND45.^21^ Under 2–3% isoflurane, a small incision was made at the level of the mid-thigh. The sciatic nerve was exposed by blunt dissection through the biceps femoris. The nerve trunk proximal to the distal branching point was loosely ligated with three 4-O silk sutures placed approximately 0.5 mm apart until the epineurium was slightly compressed and minor twitching of the relevant muscles was observed. The muscle layer was closed with 3-O silk suture and the wound closed with metal clips.

### Assessment of mechanical sensitivity

Paw withdrawal thresholds (PWT) for each animal were assessed as a measure of mechanical sensitivity. PWTs were measured prior to CCI and on days 3 and 14 post-CCI using von Frey monofilaments as previously described.^22^ Animals were placed in individual plexiglass cages on top of a wire mesh and allowed to acclimate for a minimum of 30 min. Tactile stimulation to the midplantar surface of each hind paw was performed using calibrated monofilaments with gradually increasing stiffness. Each monofilament was applied for 4–6 s in the area between the footpads on the plantar surface of each hind paw. Monofilaments with increasing force were applied until a positive response was observed (e.g., abrupt paw withdrawal, shaking, licking). When a positive response was observed, the monofilament with the next lower force was applied. The test continued 1) for 5 stimulations after a positive test was observed or 2) the upper or lower range of the von Frey monofilament set was obtained. The PWT was calculated according to the formula provided by Dixon.^23^

### Assessment of overall wellbeing

At 8 weeks of age, activity was measured in open field as a measure of overall wellbeing. This test was only performed once for each animal. Rats were placed in the middle of the open field apparatus and were allowed to ambulate for 10 min without interruption. Any-maze software (Stoelting) was used to track spatial movement in the peripheral and central zones. Distance travelled, mean speed, time immobile, zone entries and time spent in zones were collected for each rat.

### Metabolic cage system

To measure energy expenditure and related behaviors, offspring were housed individually in Promethion metabolic cages (Sable Systems International, North Las Vegas, NV) located in the Rodent Metabolic and Behavioral Phenotyping Core at the Arkansas Children’s Research Institute.^24^ This system provides continuous measurement of oxygen consumption (VO_2_) and carbon dioxide production (VCO_2_) rates, from which energy expenditure is calculated using the Weir equation.^25^ Total energy expenditure (TEE) and basal energy expenditure (BEE) were then estimated for each 12-hour light period. Respiratory exchange ratio (RER) was also calculated for defined periods as the rate of carbon dioxide production divided by the rate of oxygen consumption. Cage activity (meters travelled) was recorded in real time using multidimensional infrared beam breaks and a running wheel.

Because we were limited to 16 metabolic cages, we randomly assigned CES and STD littermates in our cohort into one of two sets of 16 animals (i.e., “Set A”, “Set B”) and measured their metabolic profiles (Fig. 1). First, when the offspring were 6 weeks of age, we collected baseline metabolic data from eight CES and eight STD offspring of equal sexes for 7 days (“Set A”; PND38 through PND45). The second set of sixteen offspring, eight CES and eight STD of equal sexes (“Set B”), received CCI surgery and were subsequently monitored in the metabolic cages continuously for 14 days after CCI (i.e., PND45 through PND59). We then returned Set A to the metabolic cages for an additional 6 days (i.e., PND59 through PND65). Wheel activity was not measured in any animal prior to CCI.

### Processing of metabolic data

Promethion Live software was used for data acquisition and processing of raw data. Data files were processed using macros to refine data into hourly averages/totals and provided circadian reports of metabolic and behavioral data for each 12-hour light/dark cycle. For each animal, data were aggregated and analysed separately in 12-hour light cycle (0700–1900) and 12-hour dark cycle (1900–0700) periods. Rates of EE were calculated by the software from VO_2_ and VCO_2_ using the Weir equation. RER is the ratio between the volume of CO_2_ being produced by the body and the amount of O_2_ being consumed and indicates whether the body is reliant more on lipid oxidation (RER ~ 0.7) or glucose oxidation (RER ~ 1.0). Cage ambulation measures fine movement and direct locomotion and excludes wheel activity. Locomotion is the sum of cage ambulation and wheel activity in meters. Human interruptions for testing mechanical sensitivity during the light cycle on days 3 and 14 led to the exclusion of these cycles from the analysis. Data obtained during the light cycle on day 10 are excluded from analysis as they were deemed outliers due to unexplained changes in the stability and amplitude of recording in all cages.

### Statistical analyses

Data were analysed using GraphPad Prism 10 (San Diego, CA) and are presented as mean ± standard deviation. Each outcome was evaluated independently for males and females. We analysed behavioral and metabolic outcomes with repeated measures analysis of variance (ANOVA) with the Geisser-Greenhous correction and post-hoc correction using the Tukey’s multiple comparisons test with individual variances computed for each comparison. Group differences were considered statistically significant at p < 0.05.

## Results

### CES increased mechanical hypersensitivity after CCI

There was a significant main effect of group assignment on PWT (F(1, 28)=177.5, p<0.0001). CES offspring exhibited a decreased PWT compared to STD offspring at 6 weeks of age (16.92 g versus 23.35 g), and on 3 days (13.14 g versus 18.14 g) and 14 days (4.6 ± 2.4 g versus 8.9 ± 2.0 g) after CCI. The main effect of sex was also significant (F(1.28)=162.9, p<0.0001) with female offspring showing lower PWT than males at all assessments (Fig. 2A–C). To determine how CES altered post-CCI PWT, we normalized the post-CCI PWT values to each animal’s baseline PWT. CES female offspring had a greater decrease in PWT 14 days after CCI than STD females (0.32 ± 0.17 versus 0.15 ± 0.05, p=0.0146) (Fig. 2C).

### CES had no impact on overall wellbeing after CCI

As expected, the body weight steadily increased from 5 to 9 weeks of age for male [F(6,36)=311.32, p<0.0001] and female offspring [F(6,30)=88.53, p<0.0001] (Fig. 3A–B). Following CCI, CES females had lower mean body weight than STD offspring. However, we found no statistically significant differences in the body weight of male or female offspring following CCI [F(1,5)=0.37, p=0.5714]. There were no differences in food (Fig. 4A–B) or water (Fig. 4C–D) consumption between groups, regardless of sex following CCI. We assessed open field at PND65 and found that CES had no effect on distance travelled, speed, immobile time, or spatial preferences in males (Fig. 5A–E) or females (Fig. 5F–J). In addition, there were no differences in these outcomes between males and female offspring.

### CES had no impact on physical mobility and activity after CCI

CCI led to an immediate decrease in activity across all subject’s post-surgery, but levels returned to baseline by the end of the study. While ambulation in the cage after CCI remained consistent throughout the 14 days post CCI (Fig. 6A–B), wheel running increased steadily over this same period (Fig. 6C–D) with females demonstrated greater ambulation and wheel running than males of both the STD group [F(1,6)=7.014, p=0.038] and CES group [F(1,6)=9.553, p=0.0214]. By 14 days after CCI, CES and STD females ran nearly five times the daily distance on the wheel compared to males (Fig. 6C–D). This difference in activity is further reflected in the combined locomotion (Fig. 6E–F) metrics of ambulation (Fig. 6A–B) and wheel usage (Fig. 6C–D). Notably, CES had no significant impact on activity measures at any point during the study (Fig. 6E–F).

### Decreased energy expenditure in male offspring after CCI

In uninjured animals, we found no difference in daily total EE between CES and STD offspring in males [PND38–45: F(1, 6) = 1.42, p>0.05; PND59–65: F(1, 6) = 2.55, p>0.05] and females [PND38–45: F(1, 6) = 1.56, p>0.05; PND59–65: F(1, 6) = 0.58, p>0.05] (Fig. 7). Similarly, we observe no difference in the basal EE between CES and STD offspring in uninjured males [PND38–45: F(1, 6) = 1.53, p>0.05; PND59–65: F(1, 6) = 3.06, p>0.05] and females [PND38–45: F(1, 6) = 2.45, p>0.05; PND59–65: F(1, 6) = 0.18, p>0.05] (Fig. 7). We consider basal EE only for the light cycle as it is during this period when animals are least active.

Following CCI, we find that male CES offspring exhibited lower basal EE [F(1, 7) = 13.29, p=0.0082] and total EE [F(1, 7) = 7.70, p=0.028] compared to STD offspring (Fig. 7A,C). However, basal EE and total EE were unchanged in female CES and STD offspring after CCI [F(1, 5) = 0.12, p>0.05 and F(1, 7) = 0.24, p>0.05, respectively] (Fig. 7B,D). Metabolic data for total EE for the dark and light cycle and the dark cycle for basal EE and basal RER can be found in Supplemental figure 1 (Supplemental figure 1A–H).

### CES had no effect on respiratory exchange ratio measures after CCI

CES did not significantly impact RER measures after CCI (Figure 7E–F). Both males and female offspring had an active RER and basal RER close to 1.0 during both dark and light cycles after CCI, indicating that carbohydrates were the primary fuel source. Interestingly, following CCI offspring in Set B had an RER similar to offspring in Set A at 6 weeks of age. However, basal RER decreased to 0.7 at 8–9 weeks of age for offspring in Set A which did not undergo CCI, suggesting the mixed utilization of carbohydrates and fat for fuel.

## Discussion

Our study is the first to report comprehensive high-resolution metabolic profiling continuously for 14 days following nerve-injury induced pain in male and female adult rats and to evaluate the metabolic effects of ELS in the setting of nerve-injury induced pain. Following CCI, both male and female rats temporarily reduced their food and water consumption and physical activity level while showing modest decreases in their EE and RER in the day following surgery. Male CES offspring significantly reduced their basal EE and total EE following CCI compared to male STD offspring which demonstrates an impact of ELS on energy metabolism after peripheral nerve injury.

Mounting evidence supports an association between rodent models of early life stress and increased mechanical sensitivity in adult offspring.^9^ ELS induced by maternal separation resulted in increased mechanical sensitivity in adult offspring of both mice ^26^ and rats.^27–29^ In rats previously exposed to ELS, mechanical sensitivity increased in inflammatory models of pain.^29,30^ Consistent with these studies, we show that adult offspring have increased mechanical sensitivity following postnatal exposure to an impoverished environment and that female offspring show an increased hypersensitivity after nerve injury compared to females that had been reared in standard housing conditions. Postnatal ELS models disrupt normal maternal care behaviors which includes the dams’ delivery of routine tactile stimulation to the offspring becomes fragmented and unpredictable. This altered stimulation occurs during a critical window of somatosensory development producing persistent effects on lifelong sensory thresholds.^31^ However, the precise mechanisms which underlie this increased mechanical sensitivity in adult offspring require further study.

The association between exposure to child maltreatment and increased rates of obesity and eating disorders in later life is well established.^32–36^ ELS impacts body weight and food consumption by altering stress-related pathways and metabolic regulation through the hypothalamic-pituitary-adrenal cortical (HPA) axis. While elevated cortisol levels promote fat storage and influence appetite-related hormones to stimulate hunger and signal satiety, respectively, the associations between ELS, body weight, and energy consumption are inconsistent in preclinical models of ELS. Ryu and colleagues reported that ELS, induced by maternal separation, promoted hyperphagia and excessive weight gain through adulthood.^36^ Conversely, maternal deprivation, where the dam is removed from the litter for longer periods than in the maternal separation model, resulted in slower weight gain and decreased food intake.^37,38^ However, an early impoverished environment, as used in the present study, was associated with reduced body weights at the end of exposure, but were equivalent at 12 months of age to offspring reared in the control environment.^20,39^ Consistent with prior findings, we found that body weight remained unchanged after CCI ^5^, but we found no differences in body weight or consumption of food and water between CES and STD offspring at 5 weeks of age in males and females. Following nerve injury, CES and STD offspring decrease in food and water consumption. These differences did not differ by group and all offspring continued to gain weight as anticipated through the end of the study at 9 weeks of age. While our study found no change in food consumption and weight gain by NLB, further research is needed to better understand the relationship between ELS, consumption, and body weight in later life.

Surrogate measures that assess the affective and emotional dimensions of pain are indispensable for the translation of preclinical observations of pain behaviors from rodents to human. The open field test is a widely used behavioral assay in rodents for assessing anxiety- and depressive-related behaviors for spatial preference as well as locomotion and general well-being.^40^ While the open field test is widely used in rodent models of ELS, a recent meta-analysis of existing studies found significant heterogeneity in their assessment of exploratory behavior with no overall change in spatial preference or locomotor activity compared to controls.^41^ Due to the cumulative effect of ELS and nerve injury, we anticipated that the CES offspring would show increased anxiety-like behaviors following CCI than offspring reared in a standard environment. However, we found no association in rodent activity or spatial preference in the open field arena across groups in males or females.

Unlike the open field test, the Promethion cage system allowed us to measure long-term activity patterns of offspring in a familiar environment which may be more sensitive to pain-related behaviors ^42^ and the effects of ELS. However, we were unable to find prior reports of how ELS impacts wheel running or cage ambulation in adult rodents. Not surprisingly, ambulatory behavior dropped precipitously following CCI surgery before returning to 50% of baseline distance. As the rats increased their wheel running steadily over the first week following CCI, the overall distance traveled exceeded baseline within 5 days of CCI. CES male rats did not appear to run as far as STD male rats, however, given the large variability in activity between animals, we didn’t have sufficient power to detect a statistically significant decrease. Consistent with abundant reports, we find that females ran significantly longer distances than males ^43,44^ regardless of group assignment.

ELS affects the hypothalamic-pituitary-adrenal (HPA) axis by altering cortisol production and disrupting metabolic regulation which alters energy balance.^45,46^ Colleulori and colleagues found that ELS by maternal separation decreased physical activity, resulting in lower EE.^47^ Conversely, in another study looking at maternal separation, no significant differences in EE were found.^48^ The literature also supports an association between nerve injury and disrupted metabolism. Following nerve injury, pain reduces physical activity which can affect energy metabolism and shift energy utilization from carbohydrate reliance to increased fat oxidation. Harris and colleagues found that EE increased following thoracic spinal cord injury despite reduced locomotor activity and decreased basal RER.^49^ Interestingly, peripheral nerve injury resulted in increased EE in female mice, but decreased EE in male mice.^50^ While others have studied the metabolic outcomes from different models of ELS, ours is the first study to examine these outcomes from ELS induced by NLB. Consistent with these prior reports, we find reduced EE in male CES rats after CCI compared to STD controls while no change occurred in females. This reduced EE may promote development of metabolic disorders observed in chronic pain patients; however, future studies are needed to explore the impact of our finding.

In conclusion, emerging evidence suggests that ELS can heighten pain sensitivity in animal models of chronic pain. However, the impact of ELS on spontaneous behaviors and metabolic functions remains poorly understood and yields inconsistent results. In this study, we provide continuous, two-week, high-resolution metabolic profiling of animal intake, physical movement, and metabolic parameters following CCI to the sciatic nerve in adult rats reared under standard or impoverished conditions. Additionally, we differentiate between dark and light cycles, which are crucial for understanding circadian rhythms in rodents. We find that ELS increases mechanical hypersensitivity following peripheral nerve injury in female offspring and decreases basal EE in male offspring after peripheral nerve injury. Our data provide valuable insights into metabolic and behavioral responses following nerve injury and contribute to understanding chronic pain development in both male and female rats.

## Supplementary Material

Supplement figure 1

## Figures and Tables

**Fig. 1.**
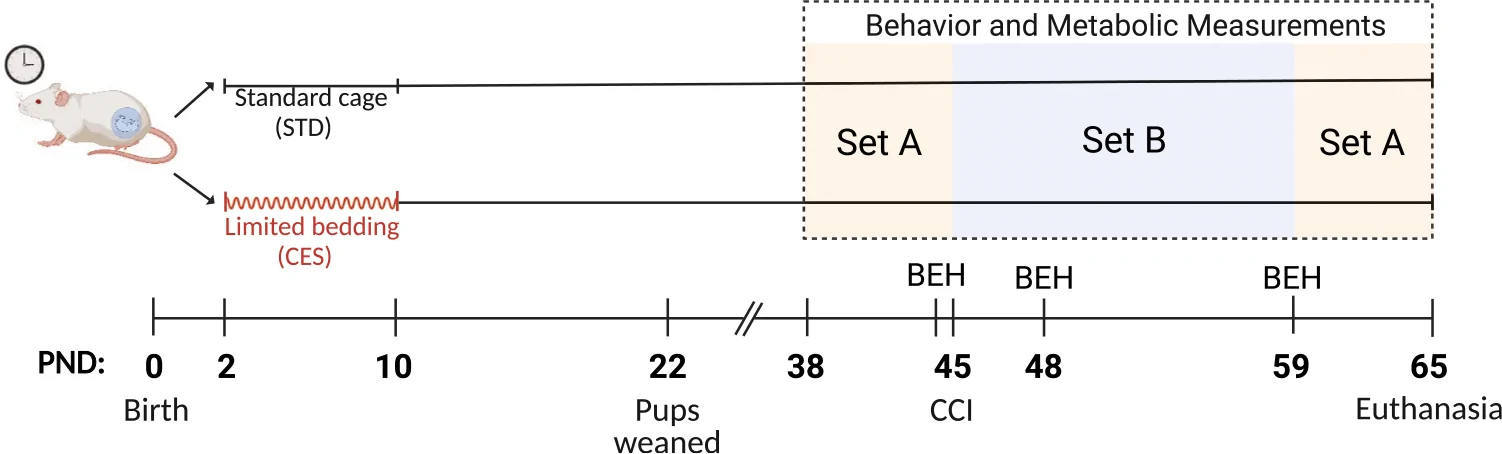
Experimental design. Litters born to timed-pregnant dams were randomized to a limited bedding or standard cage environment from PND2 through PND10, weaned at PND22, and randomly segregated to Set A or Set B for behavior and metabolic measurements. Set A (orange; n=8/group) provided control and growth-related measurements before (PND38-PND45) and after (PND59-PND65) CCI. Set B (blue; n=8/group) received CCI surgery on PND45 and were continuously monitored for from PND45-PND59. Behavior testing for mechanical sensitivity (BEH) was performed on all offspring on PND44 and on Set B on PND48 and PND59. CCI, chronic constriction injury; PND, post-natal day (age of offspring). Figure generated using Biorender.

**Fig. 2.**
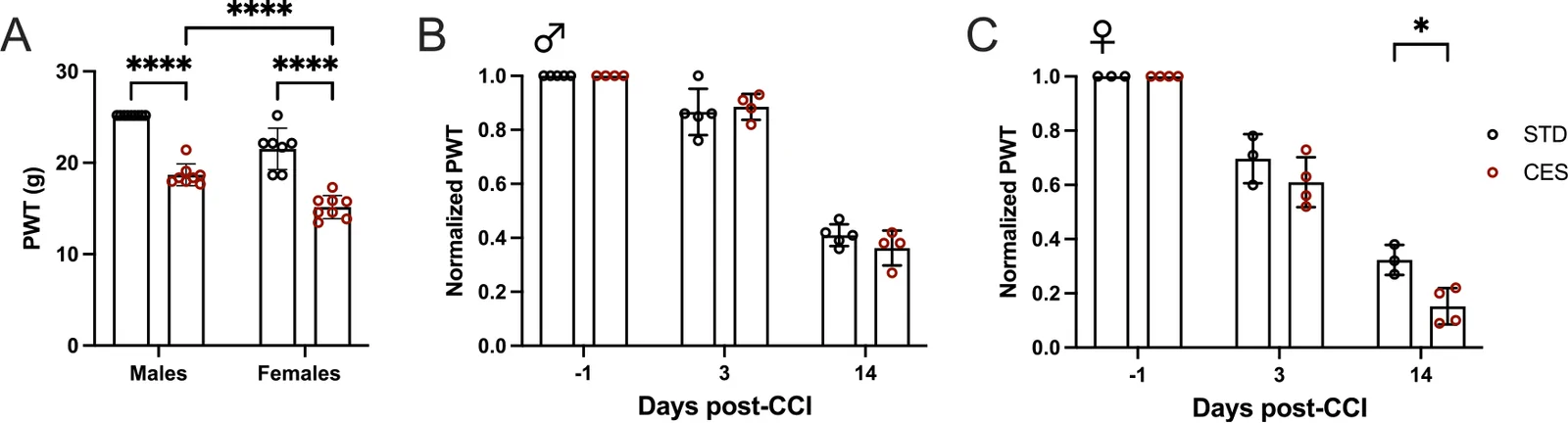
Mechanical sensitivity in STD and CES rats after nerve injury. (A) Average PWT before CCI to the sciatic nerve in STD and CES offspring (day −1), (B-C) PWT values on day 3 and day 14 post-CCI normalized to the animals baseline PWT (day −1) for (B) males and (C) and females. Data are expressed as mean ± SD. Two-way repeated measures ANOVA with main effect sex and group (A) and main effects of group and time (B and C) (*p<0.01; ****p<0.00001). CCI, chronic constriction injury; PWT, paw withdrawal threshold; SD, standard deviation; STD, standard.

**Fig. 3.**
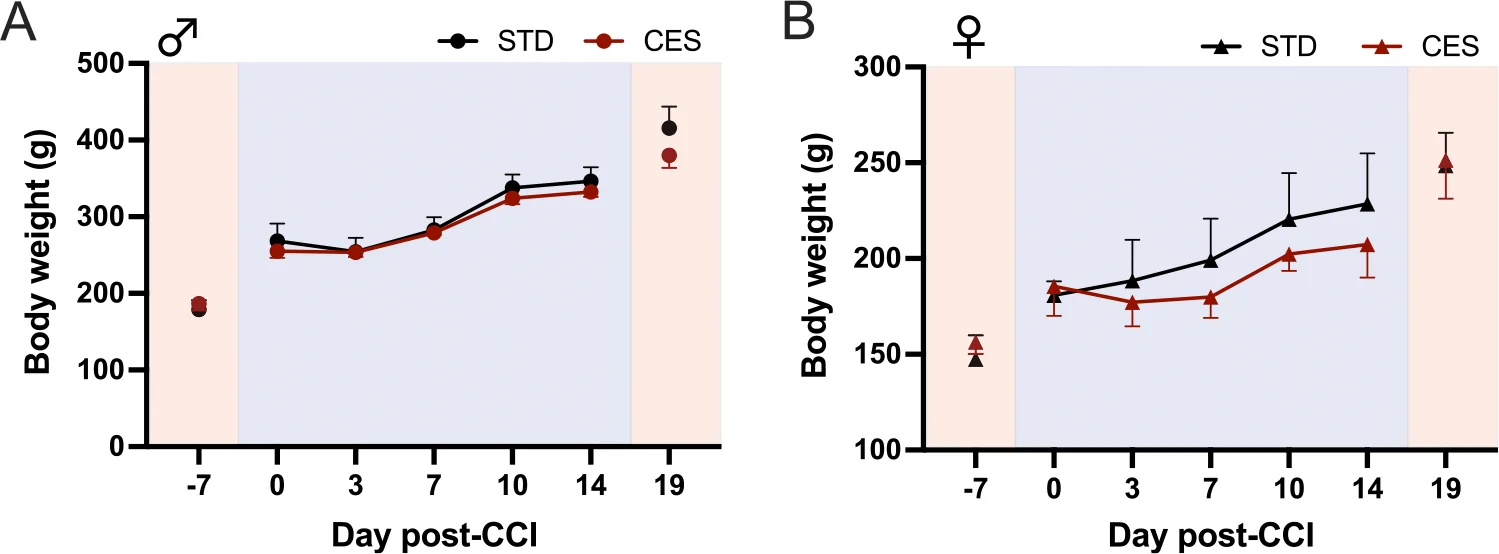
Body weight of STD and CES rats. Average body weight (grams) of (A) male offspring and (B) female offspring throughout the course of the study. Blue shaded region indicates measurements taken following Chronic Constriction Injury and orange shading highlights weight of unoperated, control CES and STD offspring. Values are mean ± SD. Significant effect of time only by two-way repeated measures ANOVA, p<0.0001 for both male (A) and female (B) offspring. CES, Chronic Early Life stress; STD, Standard; SD, standard deviation.

**Fig. 4.**
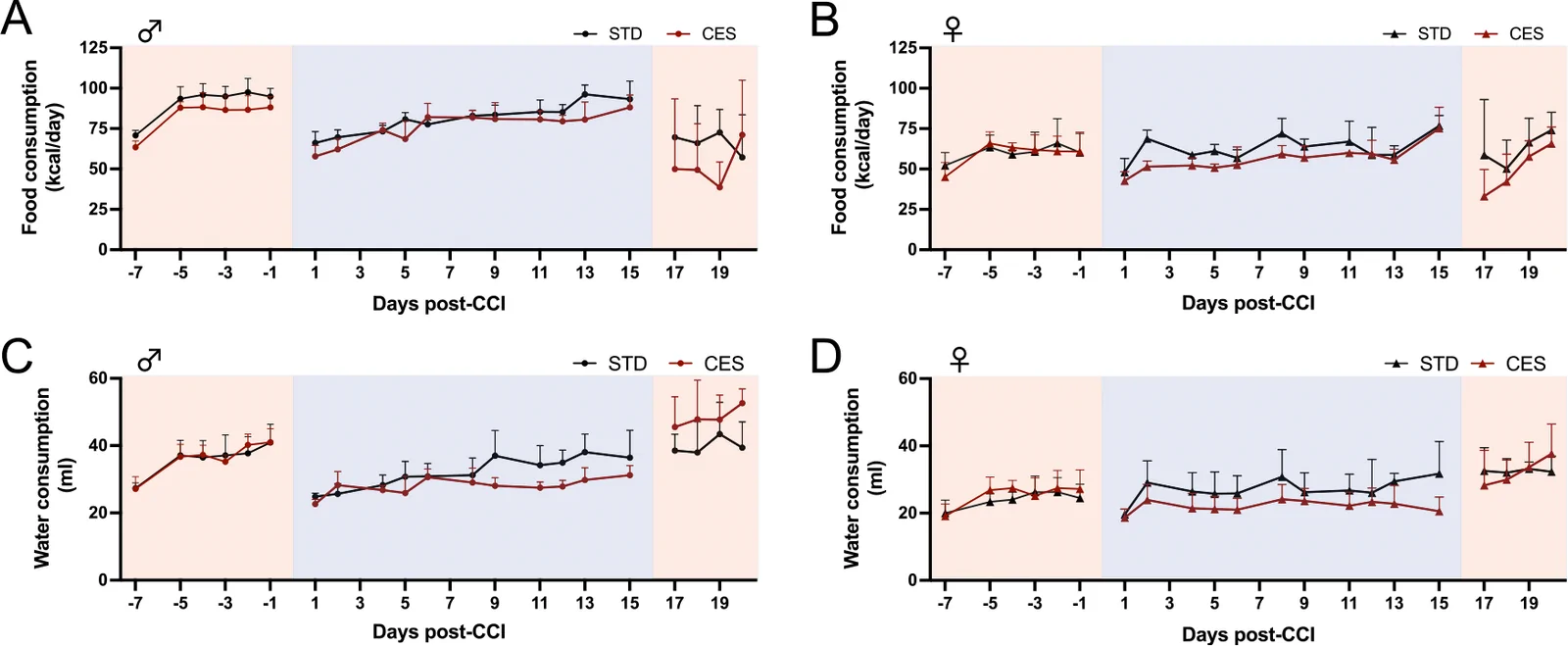
Food and water intake of STD and CES rats after nerve injury. Daily consumption of (A-B) food and (C-D) water in (A,C) male and (B,D) female CES and STD offspring. All animals had unrestricted access to food and water. Blue shaded region indicates measurements taken following CCI and orange shading highlights activity of unoperated, control CES and STD offspring. Values are mean ± SD. CCI, chronic constriction injury; CES, Chronic Early Life stress; STD, Standard; SD, standard deviation.

**Fig. 5.**
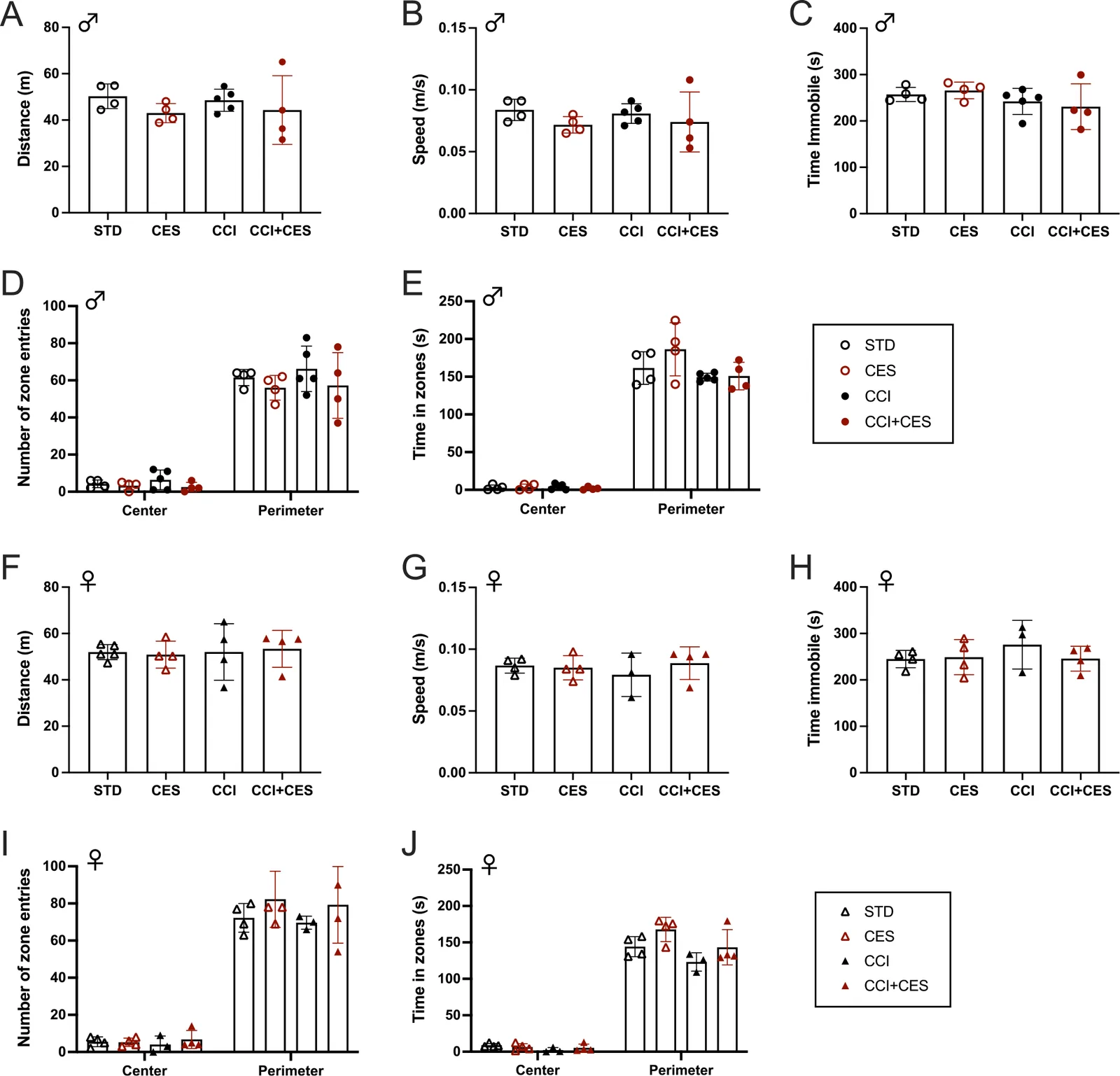
Assessment of open field in STD and CES rats. Assessments performed in once per rat at PND65 for (A-E) males and (F-J) females. Parameters include (A,F) distance travelled, (B,G) mean speed (meters/second), (C,H) seconds spent immobile, (D,I) number of entries into center and perimeter regions, and (E,J) second spent in center and perimeter regions of the open field. Values are mean ± SD. Points provide data for each individual for each parameter. CCI, chronic constriction injury; CES, Chronic Early Life stress; STD, Standard; SD, standard deviation.

**Fig. 6.**
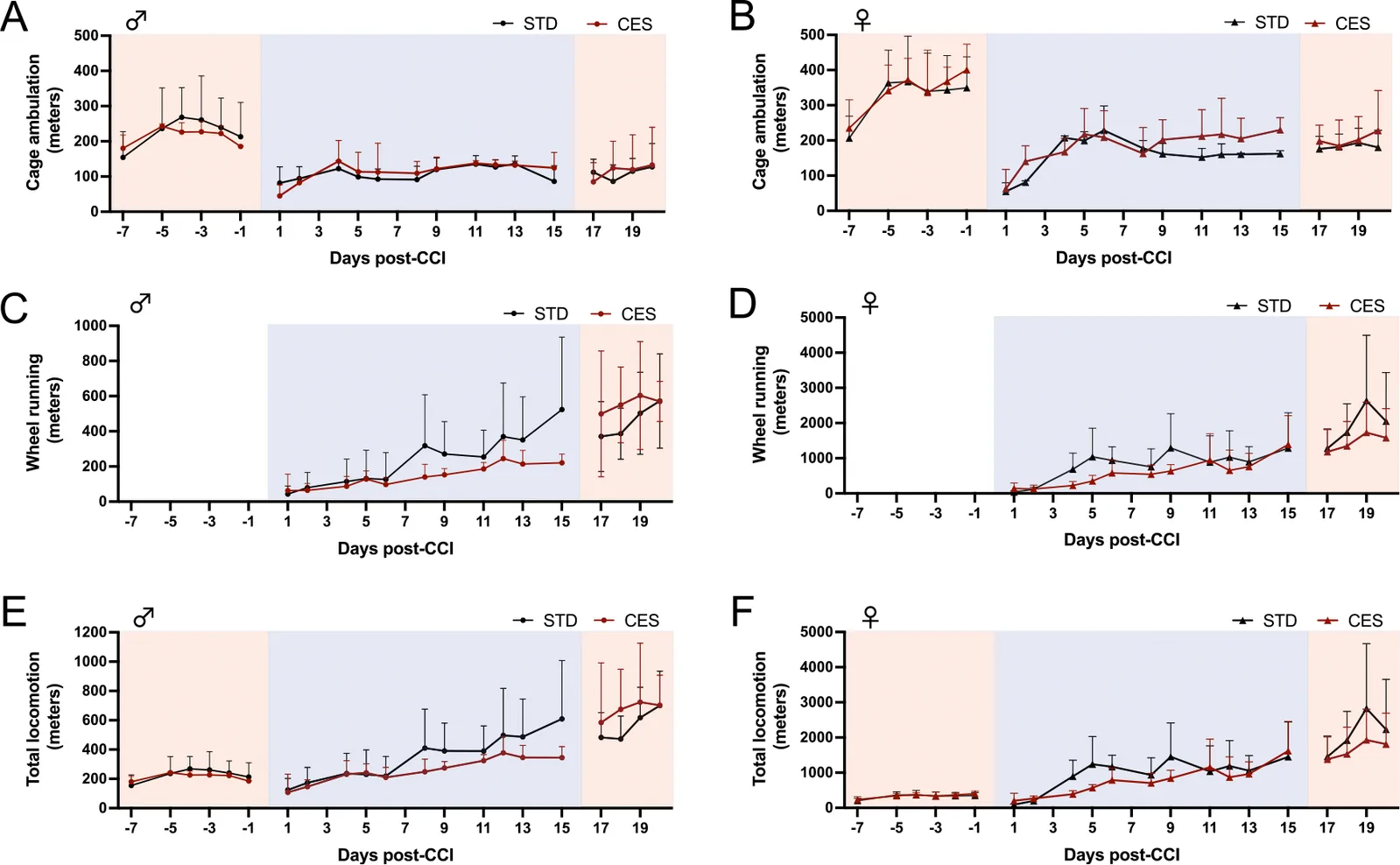
Effects of CES on motor activity after nerve-injury. Daily distance of (A-B) cage ambulation (meters), (C-D) wheel running, and (E-F) combined ambulation and wheel running for (A,C,E) males and (B,D,F) females in CES (red) and STD (black) offspring. Blue shaded region indicates measurements taken following CCI and orange shading highlights activity of unoperated, control CES and STD offspring. Wheels were available to rats only after CCI. Values are mean ± SD. CCI, chronic constriction injury.

**Fig. 7.**
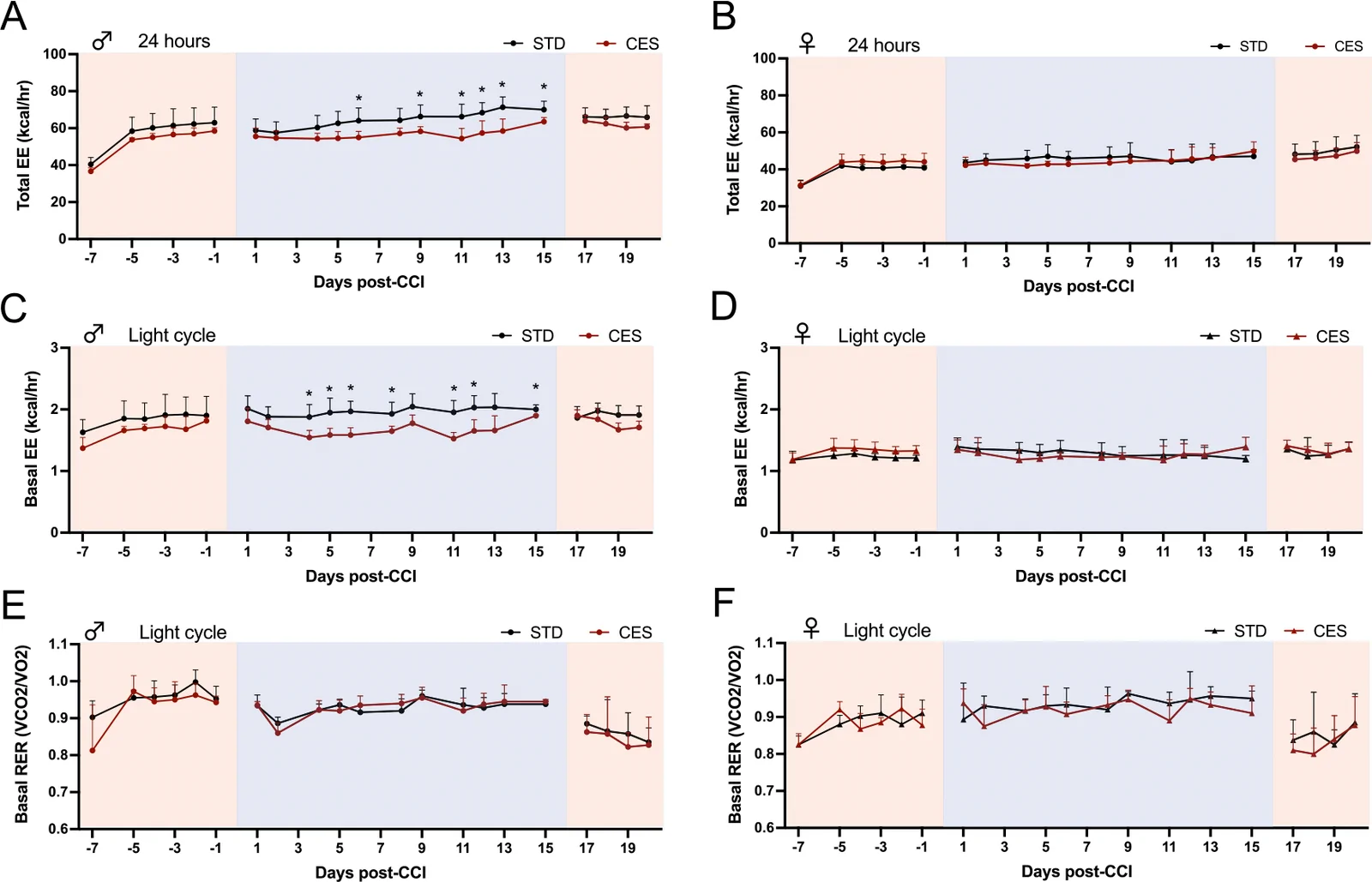
Effects of CES on energy expenditure (EE) and respiratory exchange ratio (RER) after nerve injury. (A-B) Total EE (kilocalories/hour) and (C-D) basal EE (kilocalories/hour) during daily light cycle in male (A,C,E) and female (B,D,F) CES (red) and STD (black) offspring. Blue shaded region indicates measurements taken following CCI and orange shading highlights activity of unoperated, control CES and STD offspring. (E-F) basal RER (VCO_2_/VO_2_) during daily light cycle in males (E) and females (F). Values are mean ± SD. *p<0.05 after correction for multiple comparisons. CES, Chronic Early Life stress; STD, Standard; SD, standard deviation.

## Data Availability

Raw metabolic data have been deposited at Zenodo at 10.5281/zenodo.17526996.

## Appendix A. Supporting information

Supplementary data associated with this article can be found in the online version at doi:10.1016/j.jpain.2026.106270.
