# Supplementary material for: High-resolution metabolic profiling of the effects of early-life stress after peripheral nerve injury
Source: J Pain. Author manuscript; Available in PMC 2026 Oct 4. (PMC13634588; doi:10.1016/j.jpain.2026.106270)
Supplement: Supplementary file 1 [file NIHMS2204366-supplement-Supplement_figure_1.pdf]

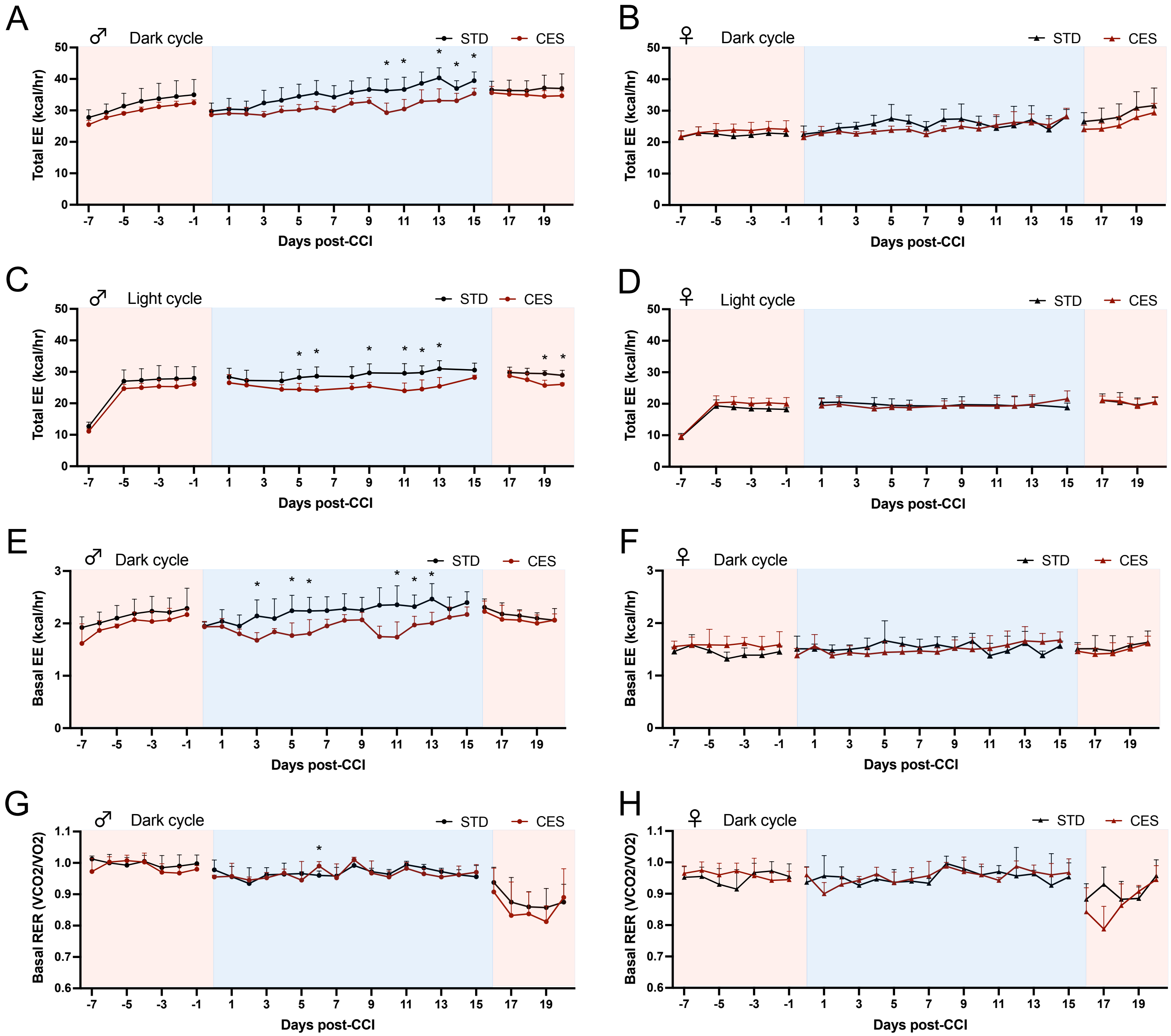

Supplemental Figure 1. Additional Data: Effects of CES on energy expenditure (EE) and respiratory exchange ratio (RER) after nerve injury. (A-D) Total EE (kilocalories/hour) during daily dark (A, B) and light (C, D) cycle in male (A,C) and female (B,D) CES (red) and STD (black) offspring. (E-F) basal RER (VCO_2/VO_2) during daily dark cycle in males (E) and females (F). (G-H) Basal RER (VCO_2/VO_2) during daily dark cycle in males (G) and females (H). Blue shaded region indicates measurements taken following CCI and orange shading highlights activity of unoperated, control CES and STD offspring. Values are mean ± SD. *$p<0.05$ after correction for multiple comparisons. CES, Chronic Early Life stress; STD, Standard; SD, standard deviation
